# Tannic Acid Modified Silver Nanoparticles Show Antiviral Activity in Herpes Simplex Virus Type 2 Infection

**DOI:** 10.1371/journal.pone.0104113

**Published:** 2014-08-12

**Authors:** Piotr Orlowski, Emilia Tomaszewska, Marianna Gniadek, Piotr Baska, Julita Nowakowska, Justyna Sokolowska, Zuzanna Nowak, Mikolaj Donten, Grzegorz Celichowski, Jaroslaw Grobelny, Malgorzata Krzyzowska

**Affiliations:** 1 Department of Regenerative Medicine, Military Institute of Hygiene and Epidemiology, Warsaw, Poland; 2 Department of Materials Technology and Chemistry, Faculty of Chemistry, University of Lodz, Lodz, Poland; 3 Faculty of Chemistry, University of Warsaw, Warsaw, Poland; 4 Department of Preclinical Sciences, Faculty of Veterinary Medicine, Warsaw University of Life Sciences, Warsaw, Poland; 5 Laboratory of Electron and Confocal Microscopy, Faculty of Biology, University of Warsaw, Warsaw, Poland; 6 Department of Morphological Sciences, Faculty of Veterinary Medicine, Warsaw University of Life Sciences, Warsaw, Poland; 7 Department of Genetics and Animal Breeding, Faculty of Animal Science, Warsaw University of Life Sciences, Warsaw, Poland; Cincinnati Childrens Hospital Medical Center, United States of America

## Abstract

The interaction between silver nanoparticles and herpesviruses is attracting great interest due to their antiviral activity and possibility to use as microbicides for oral and anogenital herpes. In this work, we demonstrate that tannic acid modified silver nanoparticles sized 13 nm, 33 nm and 46 nm are capable of reducing HSV-2 infectivity both *in vitro* and *in vivo*. The antiviral activity of tannic acid modified silver nanoparticles was size-related, required direct interaction and blocked virus attachment, penetration and further spread. All tested tannic acid modified silver nanoparticles reduced both infection and inflammatory reaction in the mouse model of HSV-2 infection when used at infection or for a post-infection treatment. Smaller-sized nanoparticles induced production of cytokines and chemokines important for anti-viral response. The corresponding control buffers with tannic acid showed inferior antiviral effects *in vitro* and were ineffective in blocking *in vivo* infection. Our results show that tannic acid modified silver nanoparticles are good candidates for microbicides used in treatment of herpesvirus infections.

## Introduction

Herpes simplex virus (HSV) causes a contagious infection affecting approximately 60% to 95% of adults worldwide. HSV-1 is associated mainly with infections of the mouth, pharynx, face, eye, and central nervous system (CNS), while HSV-2 causes infections of the anogenital area [1]–[4]. In the majority of infected individuals, HSV establishes latency in the neural ganglia, where it can be reactivated causing recurrent disease [1]–[4]. Genital HSV-2 infection is more common in women than in men. Therefore, we may assume that ulcerations resulting from recurrent HSV-2 infection consist one of the most common factors influencing integrity and functioning of the vaginal mucosa [4], [5]. At the moment, there are many experimental anti-HSV-1 and anti-HSV-2 vaccines, but none of them has been introduced into treatment of herpes infections.

Tannins are defined as high molecular weight (500–4,000 Da), polyphenolic secondary metabolites of vascular plants accumulated in leafs and fruits [6], [7]. These polyphenols are capable to form insoluble complexes with proteins, nucleic acids, carbohydrates and to chelate metal ions. Tannic acid (penta-*m*-digalloyl glucose) is the simplest and principal hydrolysable tannin. The antioxidative, anticancer and antimicrobial properties of tannic acid have been reported [8]–[14]. Lin et al. (2011) showed that hydrolysable tannins isolated from fruits of *Terminalia chebula* interact with HSV-1 glycoproteins to prevent virus attachment, entry and cell-to-cell spread [15].

Anti-bacterial and antifungal properties of silver nanoparticles (AgNPs) have been widely studied [16]–[18]. Recently, antiviral properties of AgNPs have been reported during *in vitro* studies with HIV-1 [19], [20], HBV [21], and influenza virus [22]. Lara et al. (2010) showed that AgNPs can bind to one of the HIV surface glycoprotein (gp120) and inhibit virus-to-cell attachment [20]. Baram-Pinto et al. (2009) used mercaptoethane sulfonate capped AgNPs to inhibit HSV-1 attachment to cell host membrane and thus infection [23].

Most anti-herpes drugs target the viral DNA polymerase and include a nucleoside or pyrophosphate analogues. Acyclovir (ACV), a guanosine analogue, has been the most important clinical drug for the prophylactic or therapeutic treatment of HSV infections, and represents the gold standard for the anti-HSV therapy [24]. However, extensive use of this drug has led to the emergence of ACV-resistant virus strains, particularly in immunocompromised patients [25], [26]. Recurrent herpes infections consist a particular problem in persons with immunodeficiencies, such as patients with malignancies or HIV-infected persons [27], [28]. Therefore, there is an urgent need to develop an effective anti-herpesviral microbicide.

The present study indicates that by direct blocking of viral attachment and penetration, tannic-acid modified silver nanoparticles show good anti-viral properties both *in vitro* and *in vivo*. Induction of anti-viral cytokines and chemokine production by tannic-acid modified AgNPs together with decreased inflammatory reaction *in vivo* suggests that their activity is not restricted to virus inactivation. Altogether, we show that tannic-acid modified silver nanoparticles consist a potential microbicide for herpesvirus infection in the mucosal tissues.

## Materials and Methods

### Ethics statement

This study was performed in strict accordance with the recommendations of the Polish Act of 21 January 2005 on animal experiments (OJ no. 33, item 289) and Directive 2010/63/EC of the European Parliament and the Council of 22 September 2010 on the protection of animals used for scientific purposes. The protocol was approved by the 3rd Local Committee on the Ethics of Animal Experiments in Warsaw, Poland (Permit Number: 35/2011).

### Nanoparticles

All nanoparticles used in this study were synthesized by chemical reduction method using silver nitrate (AgNO_3_) purity 99.999% (Sigma-Aldrich, St. Louis, MO, USA), sodium citrate (C_6_H_5_Na_3_O_7_*2H_2_O) purity 99.0%, (Sigma-Aldrich), tannic acid (C_76_H_52_O_46_) (Sigma-Aldrich) and sodium borohydride (NaBH_4_, purity ≥96%) (Sigma-Aldrich). Details of the synthesis method were described previously [29].

Briefly, 13 nm AgNPs were prepared as follows: into 95.5 g of the aqueous silver nitrate solution at the concentration of 0.017%, set on a mechanical stirrer, a mixture of sodium citrate (4.2 g, 4%) and tannic acid (0.6 g, 5%) was added. After mixing the reagents 0.7 g of solution of sodium borohydride, at the concentration of 2% was added. After the addition of reductants color changed to brown. The reaction was carried out for 15 minutes at room temperature, final pH value was 7.9 and the total volume of colloid was equal 100 ml.

33 nm AgNPs were prepared as follows: an aqueous solution of AgNO_3_ (95.2 g, 0.017%) was heated to boil and stirred under reflux. Afterwards, the mixture of aqueous solution of sodium citrate (4.2 g, 4%) and an aqueous solution of tannic acid (0.6 g, 5%) was added. Immediately after the addition of reductants the color of solution has changed into yellow, which indicated the formation of silver nanoparticles. The solution was vigorously stirred under reflux for additional 15 min and then cooled to room temperature. The final pH value was 6.7 and total volume of colloid was equal 100 ml.

46 nm AgNPs were prepared as described above but with the different amount of silver nitrate (98.4 g, 0.016%), sodium citrate (1.0 g, 4%) and tannic acid (0.6 g, 5%). AgNPs without tannic acid (10–65 nm AgNPs) were prepared as follows: an aqueous solution of AgNO_3_ (99.0 g, 0.0051%) was heated to boil and stirred under reflux. Afterwards, the aqueous solution of sodium citrate (1.0 g, 4%) was added. After the addition of reductants the color of solution has changed into yellow, which indicated the formation of silver nanoparticles. The solution was vigorously stirred under reflux for additional 15 min and then cooled to room temperature. The final pH value was 5.7 and total volume of colloid was equal 100 ml. It should be noted that the pH of silver colloid and biological medium was demined by medium itself for each the synthesis.

Synthesized silver colloids were examined using a Dynamic Light Scattering technique (DLS). The size and size distribution of particles in the colloids were measured using a Nano ZS zetasizer system (Malvern Instruments, Worcestershire, United Kingdom). The measured parameters were as follows: a laser wavelength of 633 nm (He-Ne), a scattering angle of 173°, a measurement temperature of 25°C, a medium viscosity of 0.8872 mPa*s, and a medium refractive index of 1.330. The samples were loaded into quartz microcuvette and five measurements were performed, for which the mean result was recorded.

### Virus

The HSV-2 strain 333 [30] was obtained from B. Adamiak, Department of Clinical Virology, Göteborg University, Göteborg, Sweden and propagated in African green monkey kidney cells (GMK-AH1) and titrated by the standard plaque assay (PFU/ml) [31].

### Cell lines

African green monkey kidney (GMK-AH1) cells were a gift from the Swedish Institute for Infectious Disease Control, Stockholm [32], mouse keratinocyte 291.03C cells were kindly provided by M. Kulesz-Martin (Departament of Dermatology, Oregon Health and Science University, Portland, OR, USA) [33]. Cells were grown in Eagle's minimum essential medium with alpha modification (α-MEM) supplemented with 10% heat-inactivated fetal bovine serum, (HI-FCS), 1% L-glutamine, penicillin (100 U/ml), streptomycin (100 µg/ml) (Gibco by Life Sciences Technologies, Carlsbad, CA, USA). 291.03C cells were also supplemented with 10 ng/ml of mouse epidermal growth factor (EGF) (Sigma-Aldrich).

### TEM and SEM images

TEM images of nanoparticles were acquired as described previously [29]. SEM images of HSV-2 interaction with AgNPs were obtained by incubating HSV-2 preparation (10^5^ PFU/ml) with 10 µg/ml of tannic-acid modified AgNPs for 30 minutes, then the mixture was added to 291.03C cell culture, incubated for 60 minutes and fixed in 2.5% glutaraldehyde in 0.1 M phosphate buffer pH 7.2 for 20 min, as described in [34] and acquired using FE-SEM Merlin (Zeiss, Jena, Germany). The elemental analysis was carried out with a multichannel EDS device XFlash Detector 5010 125 eV, Quantax Bruker (Germany) using 15 kV electron beam energy.

### Cytotoxicity

The toxicity of AgNPs and carriers was evaluated using neutral red assay. Briefly, 48 h after seeding the cells, medium was discarded and the different concentrations of nanoparticles suspended in fresh medium were added. After another 24 h, medium was collected and cells were washed with phosphate buffered saline (PBS) (Sigma-Aldrich) and fresh medium with 10% neutral red solution was then added. Cells were returned into incubator for another 2 h. Next, the cells were washed in PBS and incorporated dye was liberated from the cells according to manufacturer's protocol. Absorbance was measured at 540 nm with reference at 690 nm in Fluostar Omega microplates reader (BMG Labtech GmbH, Ortenberg, Germany). The viability of cells was expressed as the percentage of control untreated cells (100%).

### Assays for antiviral activities

291.03C keratinocytes or GMK-AH1 cells were seeded in 24-well plates (1×10^5^ cells per well) and 24 hours later infected with HSV-2 at MOI of 5. HSV-2 doses were pre-incubated, or not, with 0.5–5 µg/ml of 10–65, 13, 33 and 46 nm AgNPs or an appropriate carrier diluted in complete culture medium for 0–60 minutes. The 0 min time point means that a dose of HSV-2 was mixed with nanoparticles and immediately added to 291.03C keratinocyte or GMK-AH1 cell cultures.

At 24 h post infection (h p.i.) cells and supernatants were collected and titrated by standard plaque assay in GMK-AH1 cells to determine PFU/ml. Ten-fold dilutions of freeze-thawed lysate of cells and supernatants were utilized to inoculate GMK-AH1 cells in a 24-wells plate, and infected cells were maintained in culture for 2 days, then viral plaques were counted and expressed as PFU/ml. Each titration was done in triplicate, and experiments were repeated three times.

To analyze how AgNPs can influence viral attachment, 291.03C keratinocytes were pre-chilled at 4°C for 1 h and then co-treated with AgNPs (2.5 or 5 µg/ml) and HSV-2 (MOI of 1) for 2 h. After this time, inocula and AgNPs were removed and cell monolayers were washed with ice-cold PBS, and further incubated at 37°C. At 24 h post infection, the infected cultures were analysed by plaque assays as described above. The viral penetration assay started by pre-chilling 291.03C keratinocytes at 4°C for 1 h and then cells were infected for 2 h at 4°C to allow virus binding but not entry. The inocula were removed and cells were washed with ice-cold PBS before adding AgNPs (2.5 or 5 µg/ml) for 2 h at 37°C. The AgNPs were afterwards removed and cells were washed twice with cold PBS. After another 18 h at 37°C, cell cultures were subjected to plaque assays as described above. For examining post viral entry effects of AgNPs use, 291.03C keratinocytes were infected at 37°C with MOI of 1. Following the adsorption period, the inocula were removed, cells washed and AgNPs (2.5 or 5 µg/ml) were added at indicated time points. At 24 h post infection, the infected cultures were analyzed by plaque assays as described above. Pre-treatment was performed by incubating keratinocytes with AgNPs (2.5 µg/ml) at 37°C for 2 h, then cells were washed, infected and further titred by plaque assays.

### HSV-2 infection of mice and treatment

Female C57BL/6 mice, 6- to 8-week old, were purchased from the Mossakowski Medical Research Centre (Warsaw, Poland). Mice were injected s.c. with 2.0 mg/kg of medroxyprogesterone (Depo-Provera; Upjohn Puurs, Belgium) in 100 µl of PBS. Five days later, the mice were infected by intravaginal inoculation of 10^4^ PFU/mouse of HSV-2 strain 333 in 20 µl of PBS previously incubated for 1 h with or without 5 µg/ml of 13, 33 and 46 nm AgNPs, or a corresponding carrier buffer. To examine antiviral effect of AgNPs treatment in infected animals, at 3 or 18 h p. i. mice were irrigated 3 times with 100 µl of 5 µg/ml of 33 nm AgNPs, or a corresponding carrier buffer in PBS. At 48 h p.i. animals were sacrificed and the vaginal tissue was isolated for further preparation of histological sections or preserved in RNAlater cryoprotectant (Sigma-Aldrich) before virus titration. Experimental groups consisted of 5–6 mice, experiments were repeated three times.

### Virus titration by RT-PCR

Total DNA was isolated from the vaginal tissues preserved in RNAlater (Sigma-Aldrich) using AllPrep DNA/RNA kit (Qiagen, Hilden, Germany). HSV-2 was detected using a HSV-2 probe labeled with FAM (6-carboxyfluorescein) in a real-time PCR instrument ABI Prism 7000 (Applied Biosystems, Carlsbad, CA, USA) and titrated as described by Namvar et al., 2005 [35].

### Immunohistochemistry of animal tissues

All vaginal tissues were fixed in 4% PFA in PBS for 24 h, then processed by common paraffin technique. 5–6 µm thick vaginal sections were collected from the whole vaginal tissue every 50 µm using a rotary microtome (Leica RM 2125 RTS, Singapore). Antigens were unmasked in 0.1 M citrate buffer (pH = 6) for 10 min. HSV-2 antigens were labeled with rabbit polyclonal anti-HSV-2 (1∶250) (Dako, Glostrup, Denmark) antibody and EnVision+ System-HRP (Dako), according to manufacturer's protocol. Gr-1-positive cells (neutrophils and inflammatory monocytes) were detected using anti-Ly-6G and Ly-6C (Gr-1) antibody (RB6-8C5) (BD Biosciences, Franklin Lakes, NJ, USA) in PBS with 1% bovine serum albumin, followed by 30 minutes incubation with biotinylated anti-rat IgG antibody (BD Biosciences) and streptavidin-peroxidase (BD Biosciences) in the same buffer. Sections were developed with 3,3′-diaminobenzidine (DAB) and counterstained with Harris's hematoxylin solution (Sigma-Aldrich). Image capture, analysis and processing were performed using the Zeiss Axio Imager.M2 microscope and ZEN 2011 software (Zeiss). For all stainings, isotype control antibodies - rat monoclonal IgG or rabbit polyclonal were used (BD Biosciences). Area of an infectious site was measured using ZEN 2011 software. Numbers of Gr-1-positive cells per one HSV-2 infected site were counted as all Gr-1-positive cells, both mononuclear and polymorphonuclear, detected at the infected site within the vaginal epithelium. To avoid sampling errors, all step sections collected every 50 µm of the whole vaginal tissue were stained. The image analysis was performed independently by two persons unaware of treatment.

### Measurement of cytokines

Concentrations of cytokines in vaginal lavages were determined using Mouse Inflammation Cytometric Bead Array (CBA) (BD Biosciences) according to the manufacturer's protocol. The results were presented as the means of assays performed in triplicates. Data were analyzed by using FCAP Array 1.0.1 and BD Cytometric Bead Array 1.4 software.

### Statistical analysis

Quantitative data were presented as means ± S.E.M. For normal distribution of values, statistical comparisons were performed using the Student's t-test, data following non-Gaussian distributions were analysed with non-parametric Wilcoxon test. The number of mice per group was calculated with the power level set to 0.8 and the conventional level of alpha (0.05) for a paired t-test. Values of P<0.05 were considered significant.

## Results

### Characterization of AgNPs

**Figure 1:**
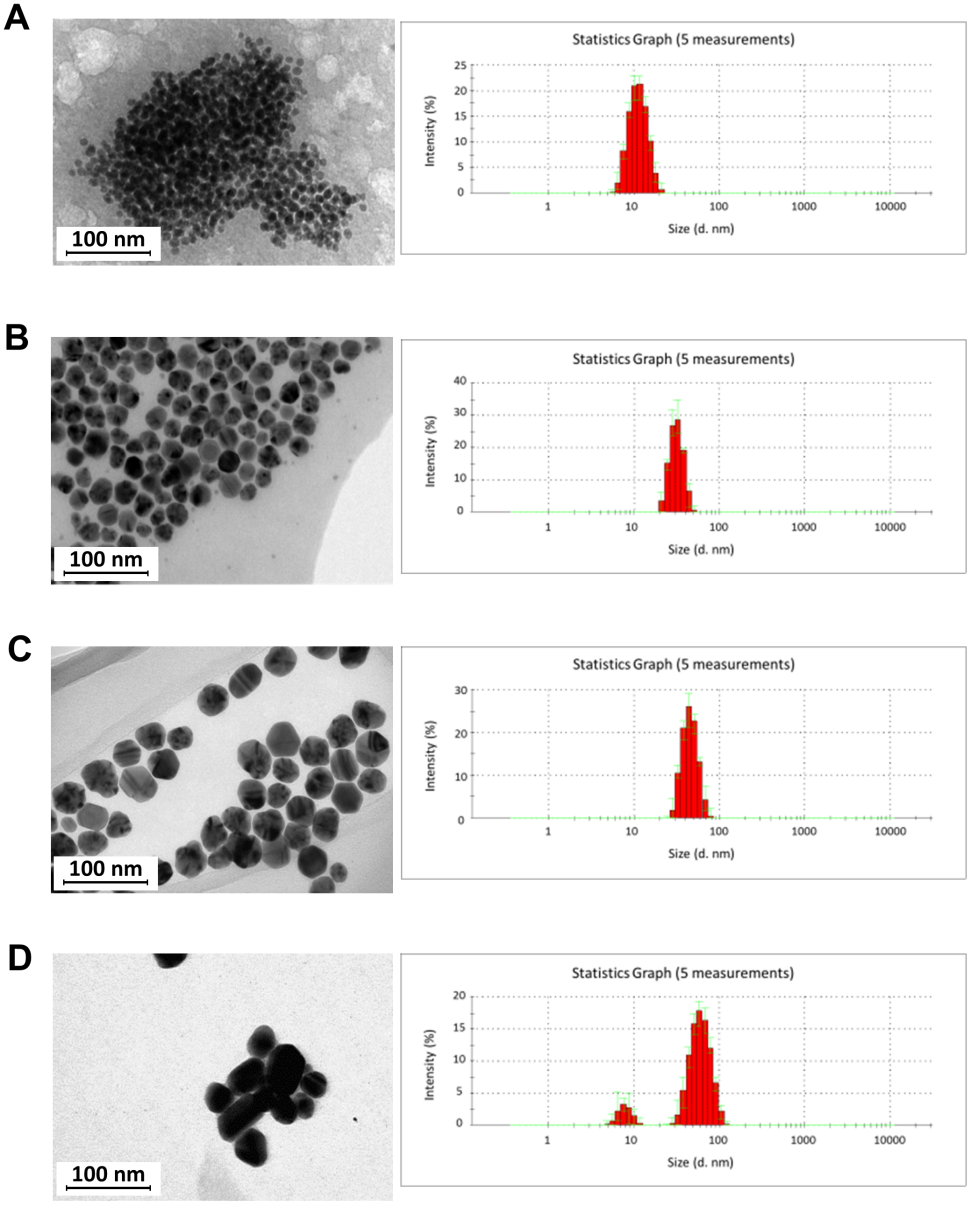
Transmission electron microscopy (TEM) images of silver nanoparticles and DLS histograms. (A) 13±5 (13) nm, (B) 33±7 (33) nm, (C) 46±9 (46) nm and (D) 10±1–65±10 nm AgNPs.

Silver nanoparticles in stock solutions were characterized by TEM and DLS technique (Fig. 1). The obtained results showed that the tested colloids contain monodisperse nanoparticles with the sizes of 13±5 nm (A), 33±7 nm (B), 46±9 nm (C) and 10–65 nm (D). Therefore, AgNPs used for further research were designed as 13 nm (A), 33 nm (B) and 46 nm (C) and 10–65 nm (D). To investigate the stability of the colloids, zeta potential was measured. The values obtained for each colloid: (A) −31±7 mV, (B) −58±2 mV, (C) −56±2 mV and (D) −64±1 mV indicate the stability of the nanoparticles over a long time. Colloids stability confirmed by a DLS test performed after one and six months from the initial date of synthesis showed that the measured size of nanoparticles was constant within the measurement error.

### Inhibition of HSV-2 infection *in vitro* by tannic acid modified AgNPs is dose and size-related

To ensure that tested AgNPs concentrations were not toxic, cytotoxicity of nanoparticles in 291.03C cell line was assessed using neutral red assay (Table 1). The 50% cytotoxic concentration (CC_50_) for 33 and 46 nm AgNPs was approx. 4 times lower than that for 13 and 10–65 nm AgNPs (Table 1). Therefore, for *in vitro* infectious assays we chose concentration of 5 µg/ml for 33 and 46 and 2.5 µg/ml for 13 nm and 10–65 nm AgNPs. The results of more detailed toxicity tests were published previously and showed that tannic acid-modified AgNPs sized >30 nm were relatively non-toxic to mouse keratinocytes [29].

**Table 1:** Cytotoxicity and anti-HSV-2 activity of tannic acid-modified 13, 33, 46 nm AgNPs, unmodified 10–65 nm AgNPs or corresponding carriers in 291.03C cells.*

AgNPs	Cytotoxic concentration Mean CC_50_ †	Antiviral effect of AgNPs	
		Mean EC_50_ ‡	SI§
	µg/ml ± SEM	µg/ml ± SEM	
13 nm	4.18±0.32	1.99±0.02	2.1
33 nm	30.452±6.7	1.52±0.18	20.03
46 nm	19.2±2.1	2.53±0.31	7.58
UN 10–65 nm	3.83±0.45	3.93±0.79	0.97
carrier 13 nm	28.54±7.08	3.75±0.21	7.62
carrier 33 nm	19.94±4.46	2.06±0.49	9.66
carrier 46 nm	16.64±2.95	3.12±0.02	5.34

* The values shown are means from three independent experiments with each treatment performed in triplicate.

† Cytotoxic effects were evaluated by neutral red assay to determine the concentration of 50% cellular cytotoxicity (CC_50_) of the tested compounds.

‡ Antiviral effects were evaluated by plaque assay to determine the effective concentration that achieved 50% inhibition (EC_50_) against HSV-2 infection.

§ SI. selectivity index.

CC_50_/EC_50_.

The colloids of tannic-acid modified and unmodified AgNPs were compared with buffers containing the same concentrations of tannic and citric acids (µg/ml) as the 13, 33, 46 and 10–65 nm AgNPs colloids (further called carriers). The carrier buffers showed toxicity at the similar level (Table 1).

**Figure 2:**
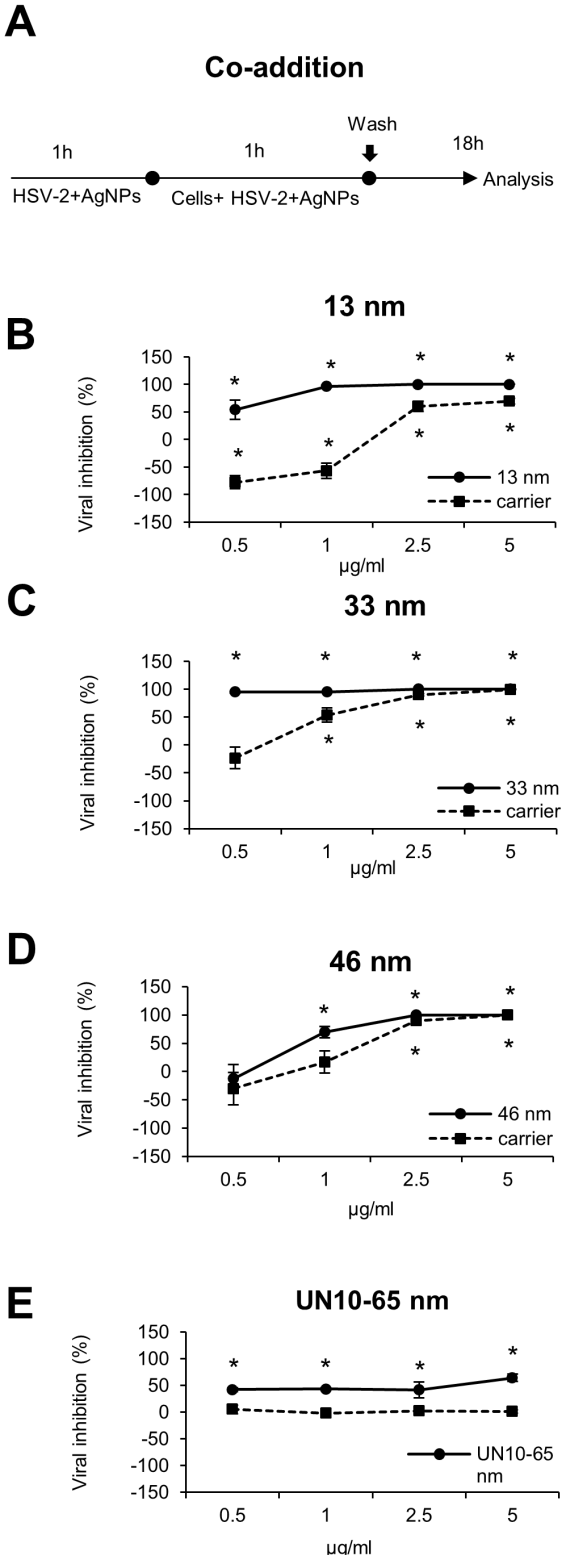
Inactivation of HSV-2 infection by tannic acid modified AgNPs is dose and size related. (A) Schematics of dose response experiments. Viral inhibition (%) in 291.03C cells infected with HSV-2 pre-incubated for 1 h with 13 nm (B), 33 nm (C), 46 nm (D) AgNPs and unmodified 10–65 nm (E) AgNPs or respective carrier buffers at 0.5, 1, 2.5 and 5 µg/ml. At 24 h p.i. cells and supernatants were collected and titrated to determine PFU/ml in comparison to HSV-2 infected cultures. The data are expressed as means from three independent experiments ± SEM. * represents significant differences with p≤0.001.

The antiviral properties of AgNPs in 291.03C cell culture were tested by standard plaque forming assay (PFU/ml). Incubation of HSV-2 with different sizes and concentrations of AgNPs for 60 minutes prior to infection showed a dose-dependent virus inactivation by tannic acid-modified AgNPs sized 13, 33 and 46 nm (p≤0.001) (Fig. 2B–D) and much less efficient virus inhibition by unmodified AgNPs sized 10–65 nm (p≤0.001) (Fig. 2E). Of all tested AgNPs sizes, 33 nm showed the highest antiviral activity already at 0.5 and 1 µg/ml in comparison to HSV-2 infected cultures (95±1.38% and 95.4±0.42%, respectively) (Fig. 2 C). In comparison to 33 nm AgNPs, similar inhibitory effect for 13 nm AgNPs was achieved at 1 µg/ml (p≤0.001) (96.86±2.05%) (Fig. 2B). For 46 nm AgNPs, 100% inhibition was observed at the concentration of 2.5 µg/ml (Fig. 2D). The lowest reduction of plaque formation was found for AgNPs without tannic acid modification - approximately 50% inhibition, irrespectively of concentration (p≤0.001) (Fig. 2E). The carrier buffers were effective only at higher concentrations, starting from 2.5 µg/ml (p≤0.001) (Fig. 2). The CC_50_/ED_50_ ratio, consisting the selectivity index (SI), showed that the most advantageous index was obtained for 33 and 46 nm (20.03 and 7.58, respectively), while 2.1 for 13 nm AgNPs. Since the SI for 10–60 nm AgNPs was only 0.97, we further focused only on the antiviral effects of tannic acid modified 13, 33 and 46 nm AgNPs (Table 1). The selectivity index for 13, 33 and 46 nm carriers was 7.62, 9.66 and 5.34, respectively (Table 1).

### Tannic acid modified AgNPs block virus attachment and penetration by direct interaction

**Figure 3:**
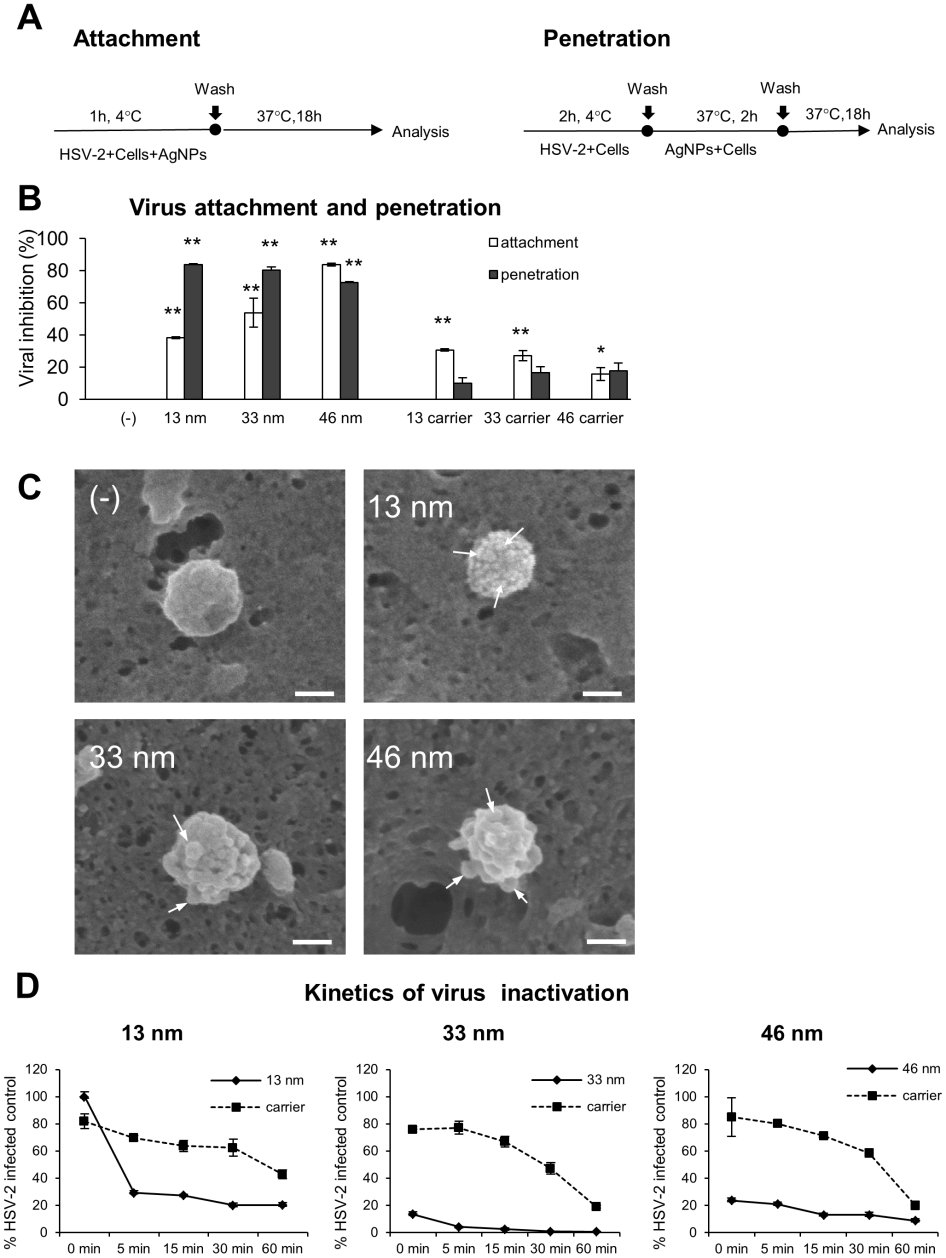
Tannic acid-modified AgNPs block HSV-2 attachment and penetration. (A) Schematics of attachment and penetration experiments. (B) Viral inhibition (%) for virus attachment and penetration experiments in 291.03C cell cultures with the use of 33 nm and 46 nm AgNPs (5 µg/ml) and 13 nm AgNPs (2.5 µg/ml) and corresponding carriers. At 24 h p.i. cells and supernatants were collected and titrated to determine PFU/ml in comparison to HSV-2 infected cultures. (C) SEM images in EDS mode of HSV-2 incubated with 13 nm, 33 nm and 46 nm AgNPs, white arrows indicate nanoparticles on the viron's surface. White bars indicate 100 nm. (D) Kinetics of AgNPs and HSV-2 interaction expressed as % of HSV-2 infected positive controls. HSV-2 aliquots were mixed with 2.5 µg/ml of 13, 33 or 46 nm AgNPs or corresponding carriers, incubated for indicated time points, then used to infect GMK-AH1 cells and determine PFU/ml in comparison to HSV-2 infected cultures. The data are shown as means from three independent experiments ± SEM. * represents significant differences with *p*≤0.05, while ** *p*≤0.001.

To understand the antiviral mechanism(s) involved, we tested the effect of tannic acid modified AgNPs against HSV-2 attachment to the host cell surface and subsequent membrane fusion. The use of different temperatures - 4°C and 37°C allows to examine the effect of AgNPs on each specific step of viral infection (Fig. 3A). All tannic-acid modified AgNPs prevented attachment of HSV-2 by 40–80%, albeit with different efficiency (p≤0.001) (Fig. 3B); the highest tested size (46 nm) being the most effective (Fig. 3B). The corresponding carriers also showed a significant inhibition of viral attachment (p≤0.05), although less efficient than AgNPs (Fig. 3B). To test if tannic acid-modified AgNPs can impair the penetration phase, HSV-2 was allowed to bind to the cell surface at 4°C and then to penetrate keratinocytes by a temperature shift to 37°C in the presence or absence of the tested AgNPs or carriers. Again, tannic acid-modified AgNPs impaired virus entry with approx. 80% efficiency, although no size-related differences were observed (p≤0.001) (Fig. 3B). The carriers did not block viral penetration in a significant manner (Fig. 3B). To understand how HSV-2 can interact with tannic-acid-modified AgNPs, we employed the SEM technique equipped with energy-dispersive X-ray spectroscopy (EDS) allowing for elemental analysis in the SEM mode. For this purpose, HSV-2 virions incubated with tannic acid-modified AgNPs were allowed to attach to the keratinocyte surface and then analysed in SEM microscope with the EDS mode. The obtained images showed that AgNPs interact with the virion's surface and in this manner nanoparticles create a physical obstacle impairing interaction with the viral receptors on the cell surface (Fig. 3C).

The kinetics of AgNPs and virus interaction (Fig. 3D) showed that process of HSV-2 inactivation by tannic acid modified AgNPs is fast and its efficiency increases with the time of treatment (Fig. 3D). HSV-2 exposure to 2.5 µg/ml of 33 and 46 nm AgNPs inhibited infection already at 0 min time point (13.62±1.6% and 23.7±1.67% of infected control, respectively) (p≤0.001) (Fig. 3D). Virus treatment with 2.5 µg/ml of 13 nm AgNPs resulted in a significant inhibition of infection after a longer, 5 min treatment (29.4±1.49%) (p≤0.001) (Fig. 3D). Again, 33 nm AgNPs showed the highest inhibition of HSV-2 infection after 60 min of incubation (p≤0.001) (0.7±0.09%) of all tested AgNPs sizes (Fig. 3D). The corresponding carriers were characterized with slower and less efficient viral inhibition during 60 minutes of incubation in comparison to AgNPs (p≤0.01) (Fig. 3D), except for 13 nm carrier at 0 minute, which inhibited HSV-2 infection more efficiently than 13 nm AgNPs (p≤0.01) (Fig. 3D).

**Figure 4:**
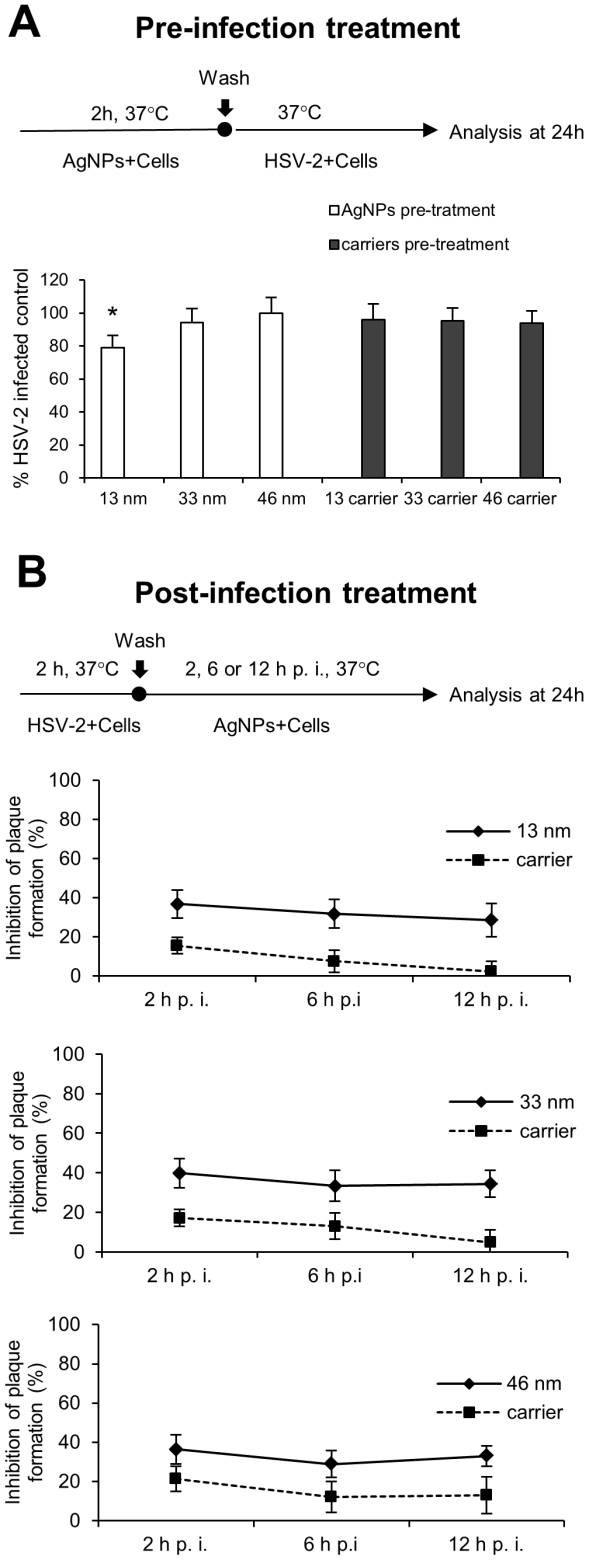
Antiviral effects of tannic-acid modified AgNPs require direct interaction. (A) Schematics and results for pre-treatment experiments. The results are expressed as % of HSV-2 infected control in 291.03C cells pre-treated with 2.5 µg/ml of tannic acid-modified 13 nm, 33 nm and 46 nm AgNPs or respective carriers for 2 h, then infected with HSV-2. (B) Schematics for post-treatment experiments. The results are expressed as percentage of viral inhibition in HSV-2 infected 291.03C cell cultures, in which complete medium containing 33 nm and 46 nm AgNPs (5 µg/ml) and 13 nm AgNPs (2.5 µg/ml) or respective carriers were added at the indicated time points for up to 24 h. The data are shown as means from three independent experiments ± SEM. * represents significant differences with p≤0.05.

To investigate the possibility of blocking cellular receptors with tannic acid modified AgNPs, we incubated 291.03C cells prior to infection with 2.5 µg/ml of 13, 33 and 46 nm AgNPs or corresponding carriers for 2 h. Incubation of the cells with 33 and 46 nm or carriers did not result in the inhibition of HSV-2 infection (94.36±8.39% and 100±9.57%, respectively) in comparison to untreated infected control (100±7.72%) (p≥0.05) (Fig. 4A). Only 13 nm AgNPs significantly decreased infection (79.17±7.4%) (p≤0.05) (Fig. 4A).

To evaluate the post-infection antiviral activity of tannic acid modified AgNPs, 291.03C cell monolayers were infected with HSV-2 and 13, 33 or 46 nm AgNPs or corresponding carriers were added after 2, 6 and 12 h of infection (Fig. 4B). Addition of tannic acid modified AgNPs starting from 2 h post infection (p.i.) led to the stable, significant antiviral inhibitory effect of approximately 30% (p≤0.05) (Fig. 4B). At the concentration of 5 µg/ml, 33 and 46 nm AgNPs but also 13 nm AgNPs at the concentration of 2.5 µg/ml exerted similar antiviral effect (Fig. 4B). The carriers significantly inhibited HSV-2 infection only when added at 2 h p.i. (p≤0.003) (Fig. 4B). These data indicate that HSV-2 infection is severely impaired if the AgNPs are present at the time of infection or at certain mechanism of viral spread (cell free virus).

### Tannic acid-modified AgNPs reduce HSV-2 infection *in vivo*

**Figure 5:**
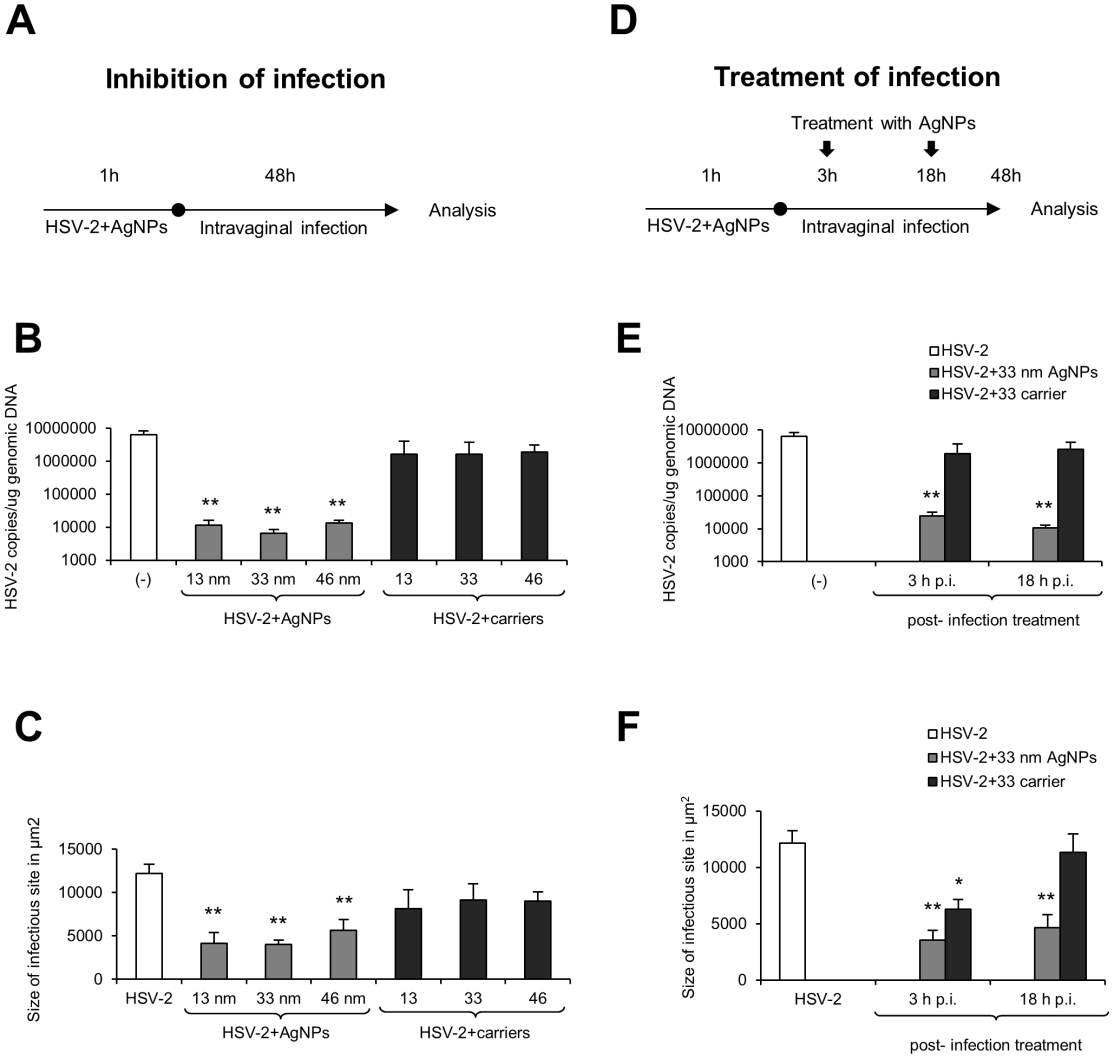
Tannic-acid modified AgNPs reduce HSV-2 infection *in vivo*. (A) Schematics of *in vivo* experiments. C57BL/6 mice were infected with HSV-2 pre-incubated or not with 13, 33 and 46 nm AgNPs or corresponding carriers (5 µg/ml). (B) HSV-2 DNA titers (copies/µg DNA) in the whole vaginal tissues determined by real-time PCR at 48 h p.i. (N = 6). (C) Sizes of infected sites determined by immunohistochemistry of HSV-2 antigens. (D) Schematics of post-infection treatment at 3 and 18 h p.i. with 33 AgNPs or carrier buffer (3×100 µl at 5 µg/ml) (N = 5). (E) HSV-2 DNA titers (copies/µg DNA) in the whole vaginal tissues determined by real-time PCR at 48 h p.i. (N = 6). (F) Sizes of infected sites determined by immunohistochemistry of HSV-2 antigens (N = 5). The bars represent means from three independent experiments ± SEM. * represents significant differences with *p*≤0.05, while ** *p*≤0.001.

To confirm our *in vitro* findings, we used the well-established murine model of HSV-2 genital infection [36]. For *in vivo* experiments, we incubated HSV-2 for 1 h prior to infection with 5 µg/ml of tannic acid modified 13, 33 and 46 nm AgNPs, then the mixtures were used for intravaginal infection of C57BL6 mice (Fig. 5A). At the peak of local vaginal infection (48 h p.i.), we observed that incubation of HSV-2 with all tested AgNPs significantly reduced viral titers measured by real time PCR in comparison to HSV-2 infected control (p≤0.001) (Fig. 5B). AgNPs of 13 and 46 nm induced a similar antiviral effect during HSV-2 infection *in vivo*, but 33 nm AgNPs were the most effective in decreasing virus titers (33 vs. 13 and 46 nm AgNPs, p≤0.05) (Fig. 5B). Pretreatment of HSV-2 with corresponding carriers did not significantly decrease HSV-2 titers *in vivo* (Fig. 5B).

Measurement of the infectious lesion areas showed that AgNPs treatment led to a significant reduction of the infected area (p≤0.001) (Fig. 5C). HSV-2 treatment with 13 and 33 nm AgNP prior to infection resulted in a similar reduction of infected areas in comparison to infected positive control (Fig. 5C). Incubation of HSV-2 with 46 nm AgNPs led to the lowest reduction of infection areas in the vaginal tissue in comparison to HSV-2 infected control (Fig. 5C). HSV-2 pre-treatment with corresponding carriers decreased the mean size of an infectious area, albeit insignificantly (Fig. 5C).

We further decided to assess the potential use of AgNPs as an antiviral agent in animals with on-going intravaginal HSV-2 infection. To study the effects of post-infection treatment, animals were infected with untreated HSV-2 and then the vaginal tissues were irrigated three times with 33 nm AgNPs or the corresponding carrier at 3 or 18 h p.i. (Fig. 5D). After 48 h p.i., the vaginal tissues were used for measurement of the virus titers and size of infectious sites (Fig. 5E and F). Post-infection treatment with 33 nm AgNPs resulted in a significant decrease of the viral titers for both time points only for AgNPs (p≤0.001) (Fig. 5E). The virus titers were significantly more reduced for treatment with AgNPs at 18 h p.i. in comparison to the treatment at 3 h p.i. (p≤0.01) (Fig. 5E). Measurement of the area of infectious sites in AgNPs treated vaginal tissue demonstrated a significant and similar decrease in the size of infectious areas at both treatment time points in comparison to infected untreated control (p≤0.001) (Fig. 5F). Treatment with 33 nm carrier resulted in a significant decrease of an infectious area size only for 3 h p.i. treatment (p≤0.05) (Fig. 5F).

### Tannic acid-modified AgNPs reduce and regulate *in vivo* inflammatory reaction during HSV-2 infection in a size-related manner

**Figure 6:**
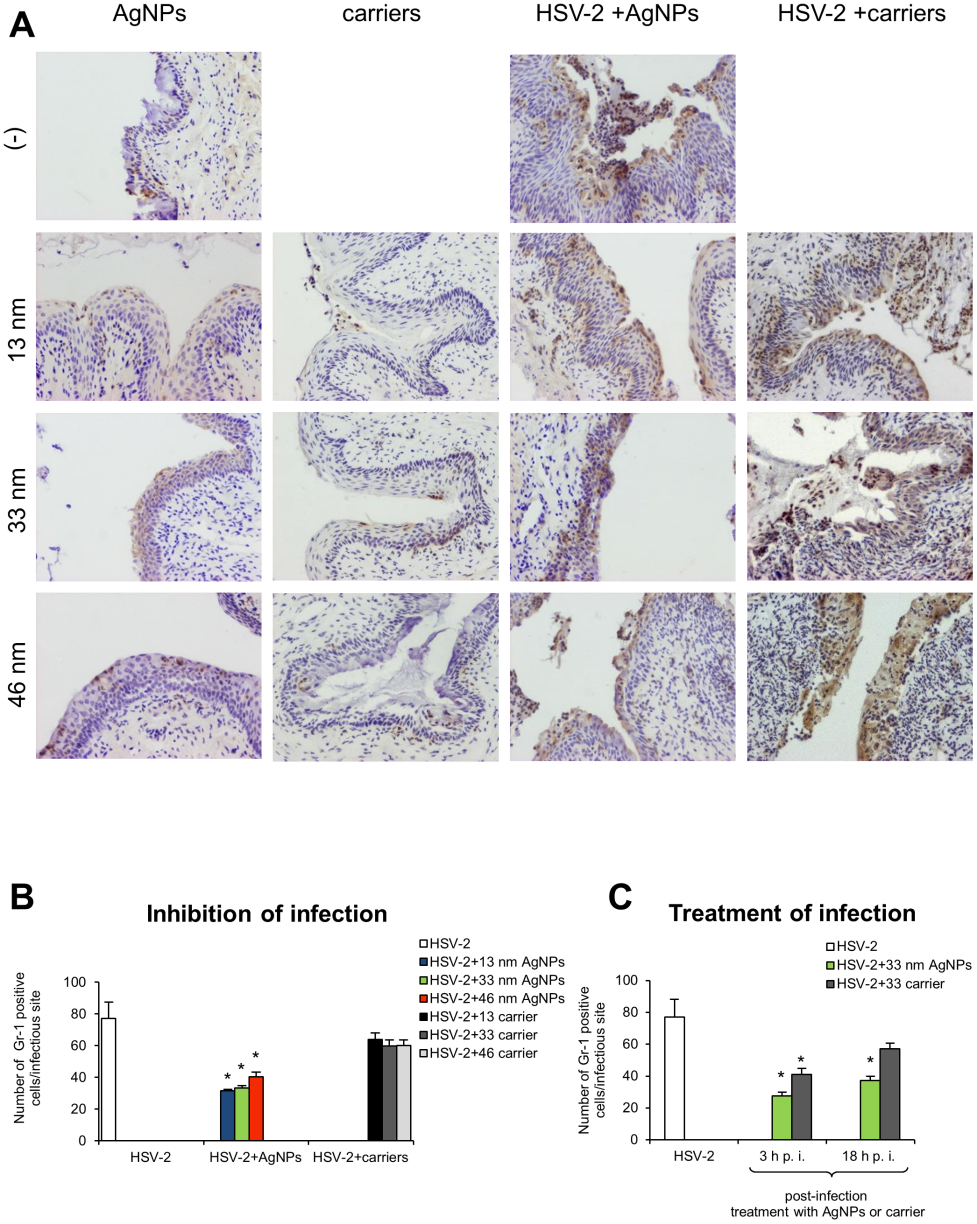
Tannic-acid modified AgNPs reduce inflammation during HSV-2 infection of C57BL/6 mice. (A) Immunohistochemical staining for Gr-1+ cells in the vaginal tissue of control mice, mice 48 h after treatment with 13, 33 and 46 nm AgNPs or corresponding carriers (5 µg/mice); mice at 48 h p.i. with HSV-2 pre-incubated with 13, 33 and 46 nm AgNPs or corresponding carriers. (B) Numbers of Gr-1+ cells/infectious site in the vaginal tissue at 48 h p.i., HSV-2 was pretreated, or not, with 13, 33 and 46 nm AgNPs or corresponding carriers. (C) Numbers of Gr-1+ cells/infectious site in the vaginal tissue at 48 h p.i. after treatment at 3 and 18 h p.i. with 33 AgNPs or the corresponding carrier buffer. The bars represent means from three independent experiments ± SEM (N = 5). * p≤0.01.

To test inflammatory properties of tannic acid-modified silver nanoparticles with or without infection, we performed staining with the anti-granulocyte receptor-1 (Gr-1) mAb, RB6-8C5 clone in (i) the vaginal tissues treated with 100 µl of 13, 33 and 46 nm AgNPs or corresponding carriers (5 µg/ml) at 48 h after treatment but also (ii) in mice infected with HSV-2 treated with AgNPs or carriers. This antibody binds to Ly6G, which is present on neutrophils, and to Ly6C, which is expressed on neutrophils, and subpopulations of monocytes (Ly6G+ and Ly6C+). Both cells populations actively participate in early inflammation during HSV-2 infection [37]. In all tested uninfected vaginal tissues, single neutrophiles in the vaginal lamina propria were observed but no significant influence upon the local inflammatory response in the vaginal tissue was detected (Fig. 6A). In the HSV-2 model, we found that pretreatment of HSV-2 with tannic acid modified 13, 33 and 46 nm AgNPs significantly reduced the numbers of Gr-1+ cells within the infectious lesions in the vaginal tissue at 48 h p.i. (p≤0.01) (Fig. 6A, B). Pretreatment of HSV-2 with corresponding carriers did not significantly decrease inflammatory reaction during subsequent infection (Fig. 6A, B). Furthermore, treatment with 33 nm AgNPs at 3 and 18 h p.i. significantly reduced the numbers of Gr-1+ cells present in the infected sites (p≤0.01) (Fig. 6 C). The carrier for 33 nm AgNPs reduced inflammatory reaction only for treatment at 3 h p.i. (p≤0.01) (Fig. 6 C).

**Figure 7:**
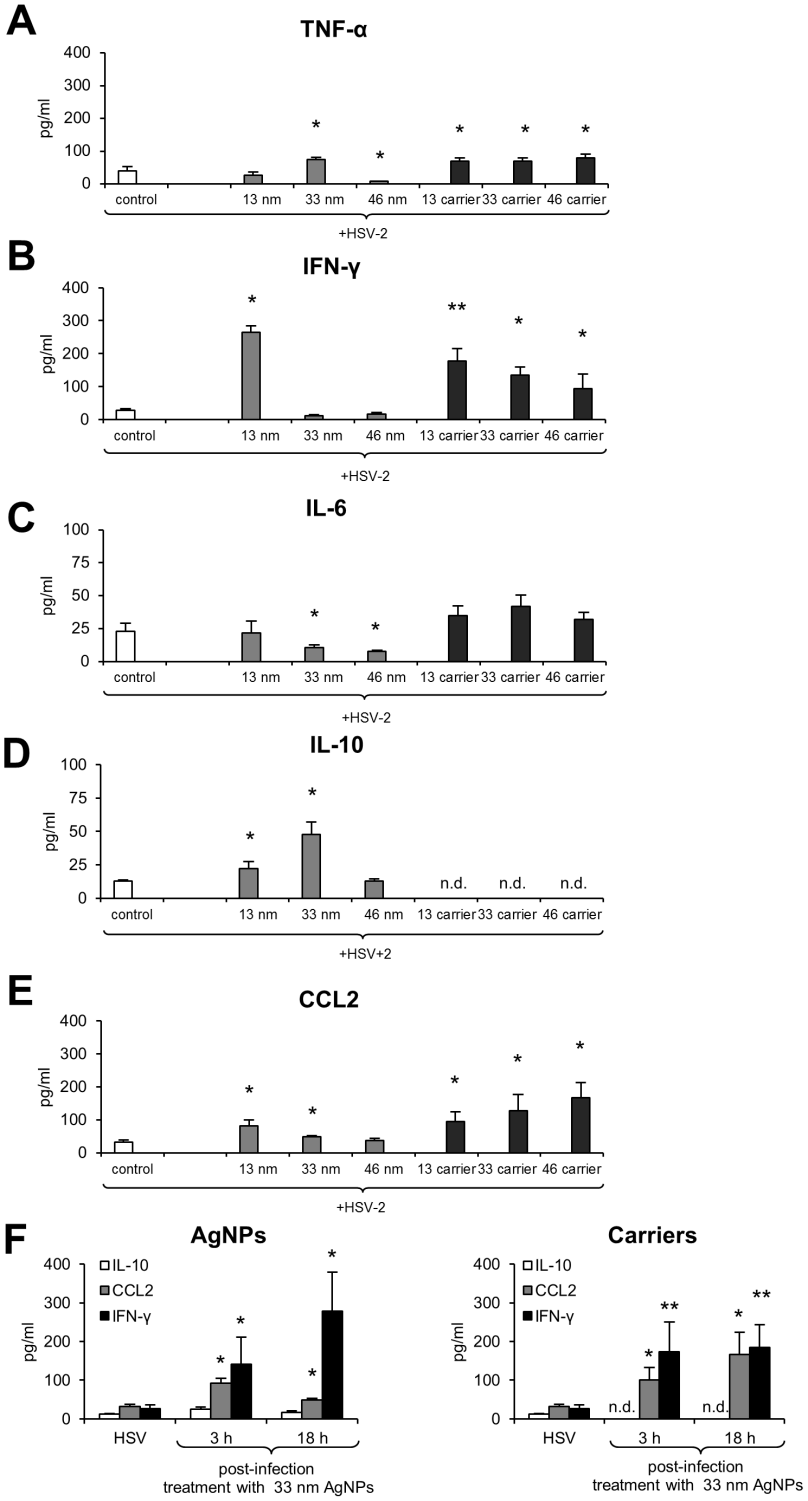
Tannic-acid modified AgNPs regulate cytokine and chemokine production in a size-related manner. TNF-α (A), IFN-γ (B), IL-6 (C), IL-10 (D) and CCL2 (E) production in the vaginal lavages of uninfected or HSV-2 infected C57BL/6 mice at 48 h with the virus dose pre-incubated or not with 13, 33 and 46 nm AgNPs or corresponding carriers. (F) INF-γ, IL-10 and CCL-2 production in the vaginal lavages from mice treated at 3 and 18 h p.i. with 33 nm AgNPs or the corresponding carrier buffer (3×100 µl of 5 µg/ml). The bars represent means from 3 separate experiments ± SEM (N = 5). * represents significant differences with p≤0.05, ** p≤0.01. n.d. - not detected.

To further check how AgNPs influence the cytokine and chemokine production during *in vivo* HSV-2 infection, we assessed the vaginal lavages for the production of interleukins (TNF-α, IL-6, IL-10, IL-12p70, IFN-γ) and CCL2 chemokine. In comparison to HSV-2 infected controls, the presence of 13 nm AgNPs at infection resulted in the significantly up-regulated production of IFN-γ, IL-10 and CCL2 (p≤0.05) (Fig. 7B, D and E). The presence of 33 nm AgNPs at infection led to significantly up-regulated TNF-α, IL-10, CCL2 and down-regulated IL-6 production (p≤0.05) (Fig. 7A, C, D, E), while 46 nm AgNPs significantly down-regulated TNF-α and IL-6 (p≤0.05) (Fig. 7A, C). No significant differences in IL-12p70 production were observed (data not shown). The pretreatment of HSV-2 with corresponding carriers significantly up-regulated production of all tested cytokines except for IL-6 and IL-10 (p≤0.05) (Fig. 7).

Interestingly, treatment with 33 nm AgNPs at 3 and 18 h p.i. up-regulated production of IFN-γ, IL-10 and CCL2 (p≤0.05) (Fig. 7F). Treatment with the corresponding carrier also led to an up-regulation of IFN-γ and CCL2 levels at both time points (p≤0.05) but no production of IL-10 was observed (Fig. 7F). No significant up-regulation of all tested cytokines and CCL2 were detected for control animals treated with AgNPs or corresponding carriers.

## Discussion

There is a strong association between bacterial and viral sexually transmitted diseases (STDs) and both the acquisition and transmission of HIV infection. Most of the evidence about the role of STDs in the transmission and control of HIV is focused on HSV-2 [28], [38]. Therefore, development of small molecules capable of inhibiting HSV-2 infection represents an attractive therapeutic strategy, particularly in immunocompromised individuals who are at risk of generating HSV-2 strains resistant to standard treatment with acyclovir. The ideal microbicides should act by blocking interactions of cell surface receptor and the virus, thereby inhibiting virus attachment/entry and possibly blocking cell-to-cell spread as well. Nanoparticle-bound ligands have potentially enhanced affinity to interact with target molecules due to their spatial orientation and large surface area.

The idea of nanoparticles, as candidate antivirals in sexually transmitted diseases such as hepatitis B virus (HBV) [21], HIV-1 [19], [20], [39], [40] and HSV [23], [39], [41] has already been explored. Mohammed Fayaz et al., (2012) tested AgNPs-coated polyurethane condom (PUC) for the ability of disrupting HSV infectivity and showed that contact of the AgNPs-coated PUC to both HSV-1 and HSV-2 was able to efficiently inactivate their infectivity in Vero E6 cells [39].

Furthermore, modified nanoparticles of noble metals have also proved their anti-viral efficiency. Polyvinylpyrrolidone coated nanoparticles (PVP-coated AgNPs) inhibited HIV-1 transmission in the explants of the human cervical culture, being a promising microbicidal candidate to prevent HIV-1 transmission *in vivo* [40]. Silver nanoparticles capped with mercaptoethane sulfonate (Ag-MES) and gold nanoparticles capped with mercaptoethane sulfonate (Au-MES) were shown to inhibit HSV-1 infection *in vitro* [23], [41].

This study aimed at assessing the antiviral effects of tannic acid modified silver nanoparticles to HSV-2 infection *in vitro* and *in vivo* at concentrations that are non-toxic to the host cells. The tannic acid modification provided the highest efficiency of inhibitory effect since silver nanoparticles without tannic acid modification (10–65 nm) did not act as quickly and efficiently as the modified AgNPs. Furthermore, our data show that viral infection was inhibited only when tannic acid modified AgNPs and HSV-2 virions directly interact with each other. Pretreatment of host cells with the tannic acid-modified AgNPs had no inhibitory effects upon HSV-2 entry. This indicates that binding of cell surface receptors or cellular entry factors for HSV-2 by the tannic acid modified AgNPs is unlikely.

HSV-2 entry and spread require a combination of viral surface proteins. The gB, gD and gH-gL heterodimer are the key HSV-2 glycoproteins for viral entry and cell-to-cell transmission in cultured epithelial cells [1]–[3]. Given the high affinity of tannins for proteins and sugars [6], [7], tannic acid can bind glycoproteins on the infectious virions making them inert, impairing glycoprotein function, and preventing successful attachment and entry of the host cell. A similar effect of blocking HSV-1 entry by hydrolysable tannins was previously showed [11], [15]. Also, the anti-viral properties of tannins may not be specific for particular viral proteins, since a broad-spectrum antiviral activity of tannins against enveloped viruses, such as human cytomegalovirus (HCMV), hepatitis C virus (HCV), dengue virus (DENV), measles virus (MV) and respiratory syncytial virus (RSV) was demonstrated [42]. Many viral glycoproteins have multiply roles, including binding to host cell receptors and mediating membrane fusion. Therefore, tannins but also tannic acid-modified AgNPs may target more than one step of infection, including virus attachment, entry and cell to cell spread.

In this study we showed that anti-viral effects of tannic-acid modified AgNPs were more efficient than the tannic acid itself in carrier buffers in terms of blocking virus attachment, penetration and cell-to-cell spread after initial infection. Furthermore, tannic acid modified AgNPs were better antivirals for *in vivo* infection, also for treatment.

We can hypothesize that tannic-acid modification of AgNPs increases biological affinity of AgNPs for viral glycoproteins, while physical size together with silver anti-viral activity enhance the total effect.

Depending on a cell type, there are two main pathways of HSV entry into host cells - non-endocytic and endocytic [1]–[3]. Nevertheless, in all entry pathways HSV utilizes a similar set of viral surface glycoproteins to enter host cells. The first step in HSV entry is the attachment of HSV through the envelope glycoproteins to receptors on the surface of the host cell [1]–[3]. This interaction allows for tight anchoring of the virion particle to the plasma membrane of the host cell, and eventually leads to membrane fusion and virus penetration into the host cell. Here, silver nanoparticles of all tested sizes blocked virus penetration in a similar manner, while blocking of virus attachment increased with size. It is possible that by interacting with the virus surface through tannic acid moieties, AgNPs create a physical obstacle impeding virus penetration (Fig. S1). The size of AgNPs may also decide about efficiency of virus - AgNPs interaction. At nano-scale, the active sites on the particle's surface increase exponentially as the particle size decreases. In other words, the surface of interaction for small nanoparticles is much higher than for bigger ones [43].

Taking into account the virus structure consisting of glycoprotein spikes with the average center-to-center spacing of 9–13 nm [44], we can expect that the larger AgNPs, the lower binding efficiency with the virion's surface (Fig. S1). The SEM images of AgNPs - HSV-2 binding (Fig. 3) confirm this suggestion showing multiply interaction of 13 nm AgNPs with the virion, while 46 nm AgNPs show much less binding. Altogether, this may explain the highest anti-viral efficiency of 33 nm AgNPs, providing the most efficient size for nanoparticle - virion interactions, which is further reflected with the most advantageous dose effect (Fig. 2) and fast kinetics of viral inhibition (Fig. 3).

Tannic acid AgNPs also exert anti-viral activity at the stage of the virus spread. Multiple spread strategies for HSVs have been described, including: transmission of free virions, movement of HSV along filopodia-like cellular membrane protrusions (surfing) towards the cell body, and lateral cell-to-cell spread [1]–[3]. The tannic acid modified AgNPs added to HSV-2 infected culture starting 2 h p.i. showed similar, albeit only approx. 30% of viral inhibition, which suggests that they are not active towards all forms of viral spread, such as for example viral surfing but it needs further clarification.

Previous studies showed that small-sized nanoparticles were more efficient in inhibiting the infectivity of analyzed viruses. AgNPs ranging from 1 to 10 nm interacted with the gp120 protein of HIV to inhibit viral attachment to host cell [19] and silver nanoparticles with a diameter of 25 nm were effective at blocking of vaccinia virus host cell penetration [45]. Silver nanoparticles produced by different fungi underwent a size-dependent interaction with herpes simplex virus types 1 and 2 and with human parainfluenza virus type 3, silver nanoparticles AgNPs sized 4–13 nm and 5–23 nm having the best antiviral activity [46]. The interactions between silver nanoparticles and viruses have been studied using spherical, uniform nanoparticles. The study by Pal et al. [2007] showed that silver nanoparticles undergo shape-dependent interaction with the gram-negative bacterium *E. coli* [47] but the exact effects of these interactions require more studies.

While 13 nm AgNPs showed good anti-viral properties at smaller concentrations, they were still toxic with a low selectivity index, understood as the ratio of inhibitory effect to toxicity (Table 1). The toxicity of small sized AgNPs must be taken into account when considering the potential use as microbicides and it has been widely studied. The *in vitro* studies revealed that AgNPs induce reactive oxygen species production leading to apoptosis or necrosis, loss of mitochondrial potential and genotoxicity in exposed cells [48], [49]. The *in vivo* toxicity assessment showed increase in platelet aggregation, accumulation of AgNPs in the liver and ability to cross brain-blood barrier [49]. The toxicity of nanoparticles is also size dependent, with the smallest nanoparticles being the most toxic ones, although the direct effect depends also on the type of affected cells. Liu et al. (2010) showed that toxicity of silver nanoparticles in four human cell lines derived from different tissues (A549, HepG2, SGC7901 and MCF-7) depended on their size, with 5 nm particles being more toxic than 20 and 50 nm [50]. On the other hand, human epidermal keratinocytes (HEKs) did not show different cytotoxicity when exposed to 20, 50 and 80 nm AgNPs [51]. We previously showed that tannic-acid modified 13 nm AgNPS were more toxic than larger sized tannic acid AgNPs [29]. Toxicity of 13 nm AgNPs was also cell-type dependent, with monocytes but not keratinocytes, producing reactive oxygen species (ROS) upon exposition [29]. Therefore, despite its anti-viral efficiency, 13 nm AgNPs are less promising as microbicides.

During HSV-2 infection, the vaginal mucosa is subjected to development of inflammatory lesions, influencing integrity of the mucosal epithelium [31]. All tested AgNPs were able to significantly decrease the virus titers and the size of infected lesions at the peak of infection (48 h p.i.). Furthermore, treatment with the 33 nm AgNPs, having the best toxicity/effectiveness ratio helped to reduce infection when applied both early and later from infection (3 and 18 h p.i.). The carrier buffers containing the same concentrations of tannic acid showed no anti-viral effect *in vivo*, while treatment worked only by reducing the size of infected lesions early during infection. These results are in contrast to many studies showing good anti-viral efficiency of tannins *in vitro* [11], [15].

Professional phagocytes (neutrophil granulocytes and monocytes/macrophages) constitute an important first line of defense against microbial intruders, including HSV-2 infection [52]. However, excessive inflammatory reaction during HSV-2 infection enlarges the size of an infected site and contributes to virus spread [37]. Therefore, an ideal anti-HSV-2 microbicide should inhibit viral infection and regulate the local inflammatory reaction at the same time. We observed that all tested AgNPs, but not corresponding carriers, significantly decreased the numbers of Gr-1-positive cells (neutrophils and inflammatory monocytes) within the vaginal tissue during HSV-2 infection, while the same doses of AgNPs did not lead to any detectable inflammatory reaction in control uninfected mice. Treatment with 33 nm AgNPs also reduced inflammation.

Silver nanoparticles have been shown to decrease the levels of inflammatory markers in wounded mice [53] and in *Chlamydia trachomatis* infected mouse macrophages *in vitro* [54]. However, studies in rats exposed to silver nanoparticles by intratracheal instillation detected increased production of IL-6, CCL2 and TNF-α in bronchoalveolar lavage fluid (BALF) [55]. We have previously described that monocytes exposed *in vitro* to tannic-acid modified AgNPs strongly down-regulated production of TNF-α, while keratinocytes showed an opposite effect of increased TNF-α production [29]. Therefore, we must take into account the fact that depending on size, toxic effects and cell type, silver nanoparticles influence inflammatory response in a different manner and the observed *in vivo* effect may consist a sum of all cell specific reactions. Here, cytokine and chemokine production during HSV-2 infection showed size-related differences for each nanoparticle type - presence of 13 nm AgNPs at the time of infection led to production of CCL2, IFN-γ and IL-10, while 33 nm AgNPs induced CCL2, TNF-α and IL-10. Larger sized AgNPs - 46 nm influenced production of only IL-6. The time of post-infection treatment also influenced the profile of produced cytokines.

TNF-α, IFN-γ, IL-6 are cytokines important for mounting a proper adaptive antiviral response to HSV-2 [56]–[58], while CCL2 can attract monocytes and T cells to the site of infection [59], [60]. TNF-α can also induce CCL2 expression and mobilize monocytes/macrophages and T cells through the chemokine receptor CCR2 [61]. IL-6 supports differentiation and proliferation of B cells and T cells [62] and both HSV type 1 and type 2 are potent inducers of IL-6 [63]. IFN-γ is required for HSV-2 clearance in the genital epithelium by CD8+ T cells and it is produced by natural killer cells early during infection [64]. The carrier buffers containing tannic acid actually increased production of TNF-α, CCL2 and IFN-γ despite lack of any viral inhibition, while only tannic-acid modified 13 and 33 nm AgNPs induced IL-10 production (Fig. 7). Interleukin-10 is an anti-inflammatory cytokine, production of which is important in mounting proper anti-HSV-2 response [65]. Although anti-inflammatory effects of tannic acid *in vitro* have been described before [14], here we did not observe reduction of inflammatory response by carriers. The early anti-HSV-2 response *in vivo* involves a plethora of cells such as neutrophils, macrophages, monocytes, natural killer cells and dendritic cells, which upon infection produce different cytokines and chemokines. Balance of the cytokine network can regulate the enhancement or inhibition of the inflammatory and adaptive immune responses. As mentioned before, the influence of AgNPs may be critical but also a complex issue, therefore it needs further studies performed both in different cell types *in vitro* and *in vivo*, during later stages of HSV-2 infection.

In conclusion, we have demonstrated that tannic acid modified AgNPs have the ability to prevent HSV-2 infection by direct inhibition of virus attachment, penetration and post-infection spread. Tannic acid used in the same concentrations shows significantly inferior antiviral effects, particularly when used *in vivo*. Therefore, tannic acid modified AgNPs can consist good candidates for effective anti-HSV-2 microbicide to be used *in vivo* due to their effectiveness at lower concentrations and induction of an anti-inflammatory response.

Since both HSV-1 and HSV-2 possess similar structure of surface proteins, tannic acid modified AgNPs can be used not only as a microbicide to treat HSV-2 infections of the anogenital area, but also to treat oral herpes infections in the form of a protective gel or cream to be applied topically.

## Supporting Information

File S1 **Schematic representation of interaction between tannic acid modified AgNPs or tannic acid and HSV-2 virion.**
(TIF)
