# Supplementary figures and images for: Tannic Acid Modified Silver Nanoparticles Show Antiviral Activity in Herpes Simplex Virus Type 2 Infection
Source: PLoS One. 2014 Aug 12;9(8):e104113. doi: 10.1371/journal.pone.0104113 (PMC4130517; doi:10.1371/journal.pone.0104113)

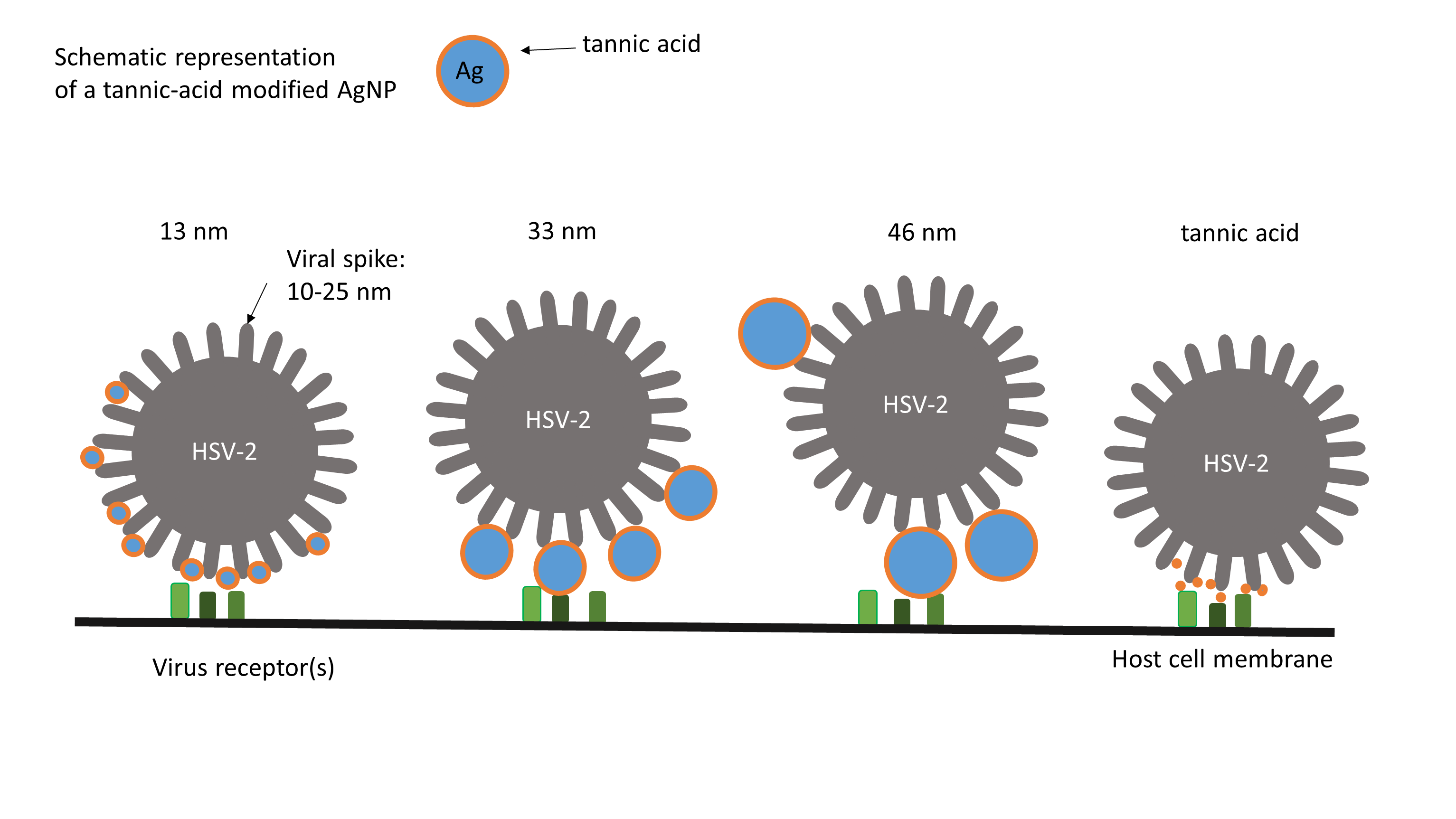

Supplement: File S1 — Schematic representation of interaction between tannic acid modified AgNPs or tannic acid and HSV-2 virion. (TIF) [file pone.0104113.s001.tif]
